# Combined protein and nucleic acid staining in tissues with PANINI

**DOI:** 10.1016/j.xpro.2022.101663

**Published:** 2022-09-09

**Authors:** Addison Deisher, Yao Yu Yeo, Sizun Jiang

**Affiliations:** 1 Center for Virology and Vaccine Research, Beth Israel Deaconess Medical Center, Harvard Medical School, Boston, MA 02115, USA; 2 Program in Virology, Division of Medical Sciences, Harvard Medical School, Boston, MA 02115, USA; 3 Department of Pathology, Dana Farber Cancer Institute, Harvard Medical School, Boston, MA 02115, USA; 4 Broad Institute of MIT and Harvard, Cambridge, MA 02139, USA

**Keywords:** Cell culture, Immunology, Microscopy, Molecular/Chemical probes, Systems biology

## Abstract

We present here a detailed protocol for PANINI (protein and nucleic acid *in situ* imaging), a technique that enables the concurrent staining of protein and nucleic acids in archival tissue sections. PANINI utilizes an optimized antigen retrieval strategy that forgoes protease treatment while retaining high sensitivity of nucleic acid detection down to single genomic events. While the protocol here is geared toward standard fluorescent microscopes with 3–4 available channels, PANINI is compatible with many commercial multiplexed tissue imaging modalities.

For complete details on the use and execution of this protocol, please refer to Jiang et al. (2022).

## Highlights

• Concurrent staining and *in situ* visualization of proteins and nucleic acids
• No protease digestion prior to ISH to maintain all protein epitopes for antibody staining
• Compatible with highly multiplexed and traditional imaging modalities
• Modular adaptability for commercial or alternative ISH assays

## Abstract (editor highlights)

Publisher’s note: Undertaking any experimental protocol requires adherence to local institutional guidelines for laboratory safety and ethics.

## Graphical abstract

**Figure:**
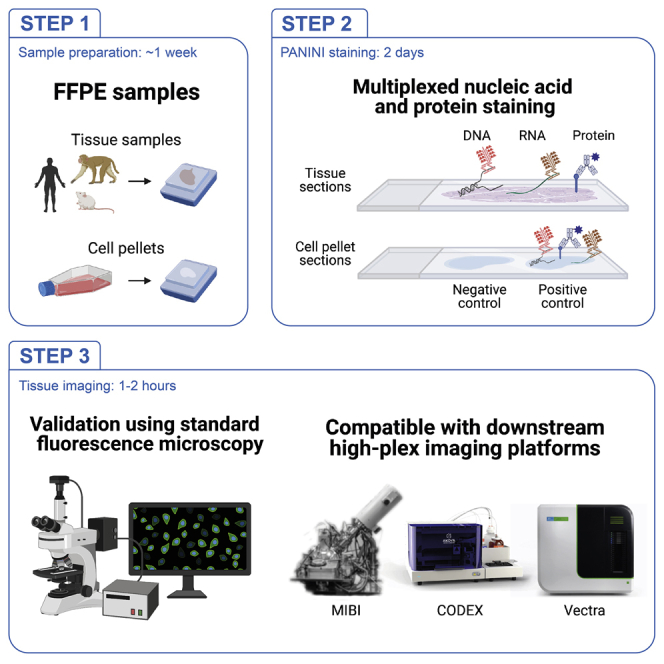

## Before you begin

The PANINI protocol prepared here enables users to visualize DNA, RNA, and proteins concurrently on a single tissue section. PANINI circumvents the standard protease treatment while retaining efficient *in situ hybridization* (ISH), thus preserving the protein epitopes for simultaneous antibody staining. We demonstrate the broad utility of PANINI by staining tissue sections from FFPE tissue blocks consisting of Simian Immunodeficiency Virus (SIV) infected and control lymph nodes from rhesus macaques, along with 3D8 (SIV positive) and CEMx174 (SIV negative) cell pellets, for SIV proviral DNA, viral RNA, and CD68+ protein.

While this protocol uses a conventional fluorescent microscope, the same workflow is applicable to downstream tissue preparation for high-dimensional imaging platforms such as MIBI (*m*ultiplexed *i*on *b*eam *i*maging) and CODEX (*c**o*-*d*etection by *i*ndexing), as demonstrated in our previous study (Jiang et al., 2022). For a detailed breakdown of the downstream staining for high-plex imaging, we refer readers to the following protocols related to CODEX (Black et al., 2021), MIBI (Elhanani et al., 2022) and CyCIF (Du et al., 2019).

### Optional (highly recommended): Cell pellet preparation

The goal of this section is to generate FFPE cell pellets that can be used for stringent validation of antibodies and hybridization probes, both essential components of the PANINI workflow. Cell lines should be carefully chosen by the user based on the study design as well as the protein/nucleic acid targets to evaluate. The cell pellet preparation assay described here is designed in concordance with tissue fixation conditions, and it is also broadly applicable to any other cell lines subjected to various genetic manipulations.

As an example, here we describe the preparation of a FFPE block consisting of 3D8 and CEMx174 cell pellets, which was used in this PANINI workflow. The 3D8 cell line was derived from a single-cell clone expansion from CEMx174 T-lymphoblast cells infected with SIV (strain SIVmac316), with each cell containing a single copy of integrated proviral DNA (Deleage et al., 2016; Nishimura et al., 2009). The paired 3D8 (SIV+) and CEMx174 (SIV-) cell pellets are therefore optimal for assessing the specificity and sensitivity of PANINI for visualizing single genomic events. Cell lines generated using transient or stable transfection/transduction of nucleic acid and protein targets have also been successfully utilized in our laboratory.

#### Part 1: Cell culture

Day 0.

**Figure 1:**
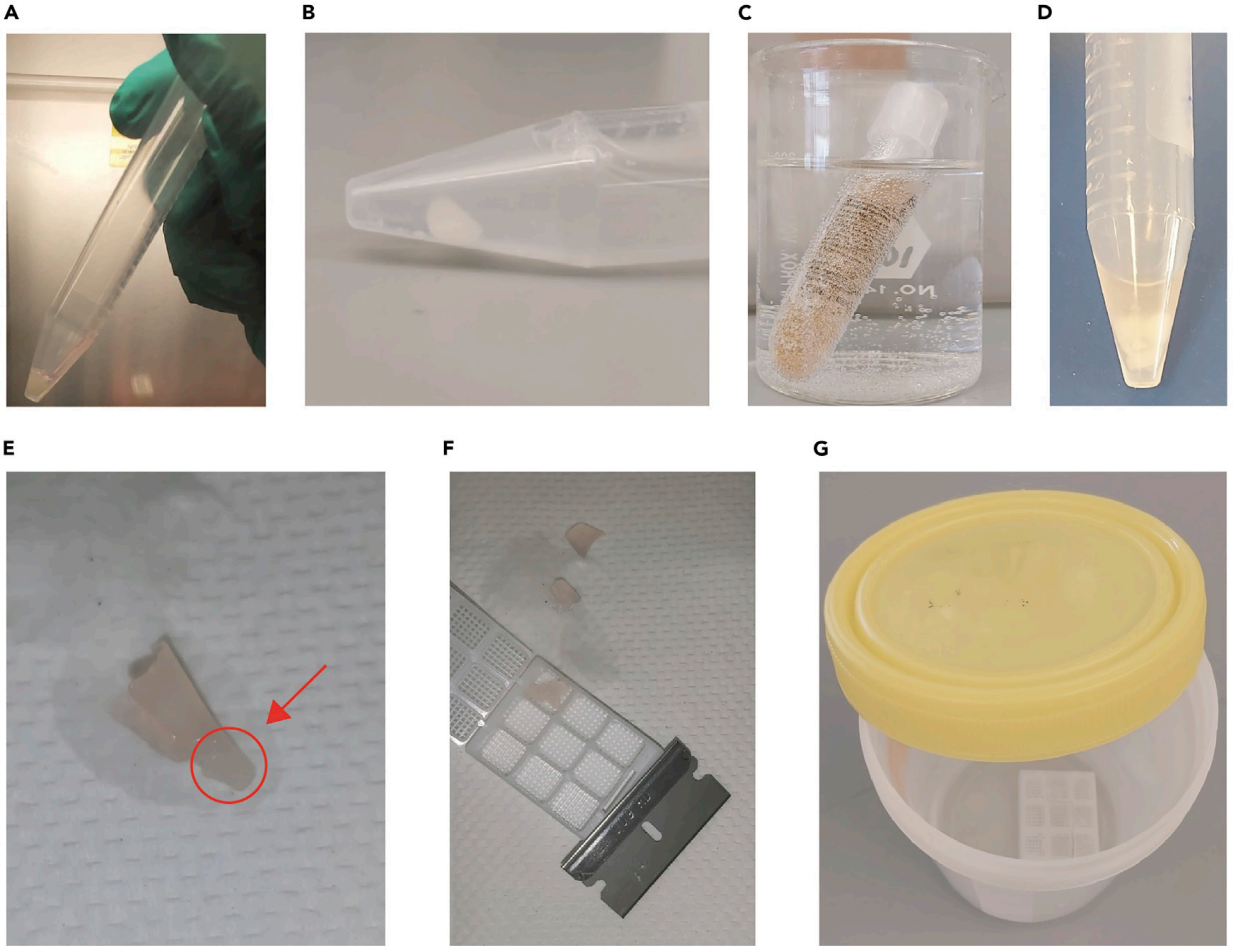
Overview of cell pellet preparation (A) Pelleted cells were harvested at 48 h post-seeding and centrifuged in a 15 mL conical tube. (B) The cell pellet was fixed with 4% PFA in 1× PBS while ensuring that it is always fully submerged. (C) The HistoGel tube was placed in a beaker of water and microwaved at 10s intervals until it completely liquified. (D) The cell pellet was resuspended with 250 μL of HistoGel by first pipetting into the bottom of the pellet, before covering the entire pellet. (E) The cell pellet (circled in red) was coagulated in HistoGel; excess HistoGel can be seen surrounding the cell pellet. (F) Excess HistoGel was trimmed off, and the cell pellet placed into a 9-compartment tissue cassette. (G) The tissue cassette was fully submerged in 75% ethanol and left at 4°C for at least 48 h.

1. Culture enough cells to make a cell pellet that is visible to the eye and of sufficient size for paraffin embedding. Since cell size varies with cell line, we recommend culturing enough cells to get pellets at least the size shown in Figure 1A. In our experience, 3D8 and CEMx174 suspension cells require at least 10 million cells to achieve this.

#### Part 2: Cell harvesting and fixation

Day 1: ∼10 min + at least 48 h incubation.

2. Collect cells into 15 mL or 50 mL conical tubes and centrifuge at 400 × *g* for 4 min at room temperature.

***Optional:*** When resuspending adherent cells, trypsin may be used but it should only be done at discretion to ensure that it does not interfere with any target surface proteins. We would suggest adhering to the cell-harvesting conditions optimized by the users for their preferred cell lines.

3. In a separate 50 mL conical tube, prepare the Fixation Solution [refer to recipe table] containing 4% PFA in 1× PBS.

***Note:*** It is important to ensure that the Fixation Solution is at a final 1× PBS concentration to prevent cell lysis.

4. After centrifugation, aspirate the supernatant and add Fixation Solution such that it fully covers the cell pellet during step 5 (see Figure 1B).

***Note:*** The cell pellet should be kept intact during this step if possible.

5. Place the conical tube on a rocker set to ∼60 rpm and ensure that the cell pellet is fully submerged in the Fixation Solution. Allow the cell pellet to fix for at least 48 h at room temperature in the dark. This fixation time can be adjusted to match the tissue fixation conditions for the tissues intended for further studies.

***Note:*** Do not rock at high speeds to prevent mechanical cell lysis.

#### Part 3: Cell pellet embedding

Day 4: ∼1.5 h + at least 48 h incubation.

6. Warm the HistoGel in a 60°C water bath until it is in a liquid state. This can be accelerated by placing the tube of HistoGel in a beaker of water and microwaving at 10s intervals (see Figure 1C).

***Note:*** Be careful when microwaving the HistoGel as once it gets hot, the cap may pop, causing the HistoGel to spill and scald.

7. Centrifuge the cell pellet at 400 × *g* for 4 min at room temperature, then aspirate the supernatant.
8. Wash the cell pellet with room temperature 1× PBS. It is not necessary to resuspend the cell pellet during the wash.
9. Centrifuge the cell pellet at 400 × *g* for 4 min at room temperature, then aspirate the supernatant.
10. Gently resuspend the cell pellet by pipetting HistoGel into the base of the pellet, then add just enough HistoGel to cover the whole pellet (see Figure 1D).

***Note:*** The pellet will break up slightly, but do not homogenize it in the HistoGel.

11. Place the HistoGel cell pellet in an ice bucket and let it solidify for at least 1 h. It will coagulate into a jelly-like cell pellet.
12. Remove the jelly-like cell pellet onto a paper towel (Figure 1E). Carefully trim the corners with a razor to remove excess HistoGel, but do not disrupt the cell pellet (Figure 1F).
13. Place the trimmed jelly-like cell pellet into a 9-compartment tissue cassette. We recommend using multi-compartment tissue cassettes as it enables the placement of different cell pellets within the same tissue blocks to help facilitate probe validation. Take note to place them in a non-symmetrical fashion to allow unique identification of each cell pellet by its location on the block, even if it is rotated.

***Note:*** If users wish to label the tissue cassette, only use a pencil because ink will get washed off by alcohols and xylenes.

14. Incubate the tissue cassette in a container containing 75% ethanol [refer to the Recipes Table] at 4°C (Figure 1G). Ensure that the container is securely sealed.

***Note:*** Any type of container can be used so long as it is securely sealed to prevent the 75% ethanol from evaporating. Cassettes can be stored for weeks in 75% ethanol.

15. The tissue cassette is now ready for processing and eventual paraffin embedding. This should be performed at a centralized histology core found at most institutions.
16. The processed paraffin blocks can then be sectioned onto slides or coverslips, which is generally performed at a centralized histology core found at most institutions. Each section should be 4–5 microns thick.

## Key resources table

**Table:** 

REAGENT or RESOURCE	SOURCE	IDENTIFIER
**Antibodies**		

anti-CD68 rabbit D4139C	CST	76437S
anti-DIG mouse 21H8	Abcam	ab420
anti-Mouse Alexa Fluor® 555	BioLegend	405324
anti-Rabbit Alexa Fluor® 488	BioLegend	406416
Streptavidin Alexa Fluor® 647	BioLegend	405237
SIVmac239-gag-pol sense (vDNA) probe	Advanced Cell Diagnostics	416141
SIVmac239-vif-env-nef-tar (vRNA) probe	Bio-Techne	416131-C2

**Chemicals, peptides, and recombinant proteins**		

**Paraformaldehyde 16% solution, EM grade**	EMS	15710
HistoGel	Thermo Fisher Scientific	HG-4000-012
Ethanol, Absolute (200 Proof), Molecular Biology Grade, Fisher BioReagents™	Thermo Fisher Scientific	BP2818100
**Xylenes (Histological Grade)**	Sigma-Aldrich	534056-500
Target Retrieval Solution pH 9	Agilent (Dako)	S2375
Gibco PBS pH 7.2 (10×)	Thermo Fisher Scientific	70013-032
ImmEdge Pen	Vector Laboratories	H-4000
RNAscope Wash Buffer (50×)	Advanced Cell Diagnostics	320058
RNAscope Hydrogen Peroxide	Advanced Cell Diagnostics	322335
20× TBS IHC Wash Buffer with Tween 20	Cell Marque	935B-09
**Borate**	Sigma-Aldrich	B6768-500G
Sodium Hydroxide	Sigma-Aldrich	S5881-500G
Dextran Sulfate 50% Solution	EMD Millipore	S4030
**Hydrogen Peroxide Solution 30%**	Sigma-Aldrich	H1009-100mL
TSA Plus Biotin 50-150 slides	Akoya	NEL749A001KT
TSA Plus DIG, 50–150 slides	Akoya	NEL748001KT
Bovine Albumin (BSA)	Thermo Fisher Scientific	BP1600-100
Donkey Serum	Sigma-Aldrich	D9663-10mL
10% Triton X-100	Sigma-Aldrich	T8787-250mL
**Sodium Azide**	Sigma-Aldrich	S8032- 25G
Hoechst 33342 Trihydrochloride, trihydrate	Thermo Fisher Scientific	H3570
ProLong™ Gold Antifade Mountant with DAPI	Thermo Fisher Scientific	P36935
Invitrogen UltraPure Distilled Water	Thermo Fisher Scientific	10977-015

**Critical commercial assays**		

Avidin / Biotin Blocking System	BioLegend	927301
RNAscope Multiplex Fluorescent Detection Reagents v2	Advanced Cell Diagnostics	323110
RNAscope 2.5 HD Detection Reagent- BROWN	Advanced Cell Diagnostics	322310

**Other**		

9-compartment biopsy cassettes	EMS	70061-03
Nail polish	EMS	72180
VWR micro cover glass No. 1, 22 × 50 mm	VWR	48393-059
Small Cell Scraper	Corning	3010
Fisherbrand™ Superfrost™ Plus Microscope Slides	Thermo Fisher Scientific	22-037-246
Heratherm OGS60 Lab Oven	Thermo Fisher Scientific	51028112
Leica ST4020 Small Linear Stainer	Leica	14050946425
Thermo Scientific™ Lab Vision™ PT Module	Thermo Fisher Scientific	A80400012
HybEZ™ II Hybridization System for Manual Assays and Pan	ACD Bio	310013
Keyence BZ-X810	KEYENCE	BX-X810
Coplin jars	Bel-Art	F44208-1000
Glass Coplin jar	Thermo Fisher Scientific	E94
Slide chamber for retrieval	EMS	62705-01
Staining tray with attached handle	EMS	72220-07
EasyDip™ Slide Staining Kit (Jar+Rack)	EMS	71388-01

Biological Samples		

3D8	HIV Reagent Program	ARP-13239
CEMx174	ATCC	CRL-1991

## Materials and equipment

**Table:** Optional Equipment

Equipment	Manufacturer	Catalog #
Heratherm OGS60 Lab Oven	Thermo Fisher Scientific	51028112
Leica ST4020 Small Linear Stainer	Leica	14050946425
Thermo Scientific^TM^ Lab Vision^TM^ PT Module	Thermo Fisher Scientific	A80400012
HybEZ™ II Hybridization System for Manual Assays and Pan	ACD Bio	310013
Keyence BZ-X810	KEYENCE	BX-X810
Coplin jars	Bel-Art	F44208-1000
Glass Coplin jar	Thermo Fisher Scientific	E94
Slide chamber for retrieval	EMS	62705-01
Staining tray with attached handle	EMS	72220-07
EasyDip™ Slide Staining Kit (Jar+Rack)	EMS	71388-01

### Buffer recipes

Use caution when making buffers as some chemicals are hazardous. Be sure to check the SDS beforehand and wear proper PPE.

**Table:** 80% Ethanol (v/v)

Reagent	Final concentration	Amount
100% Ethanol	80% (v/v)	800 mL
ddH_2_O	N/A	200 mL
**Total**	**80% (v/v)**	

Store at RT up to 6 months.

**Table:** 70% Ethanol (v/v)

Reagent	Final concentration	Amount
100%Ethanol	70% (v/v)	700 mL
ddH_2_O	N/A	300 mL
**Total**	**70% (v/v)**	

Store at RT up to 6 months.

**Table:** 1× pH 9.0 Dako Retrieval Buffer

Reagent	Final concentration	Amount
10× pH 9.0 Dako	1×	9 mL
ddH_2_O	N/A	81 mL
**Total**	**N/A**	**90 mL**

Make fresh for each use.

**Table:** 1× PBS

Reagent	Final concentration	Amount
10× PBS	1×	100 mL
ddH_2_O	N/A	900 mL
**Total**	**N/A**	**1,000 mL**

Store at RT for up to 1 year.

**Table:** 1× RNAscope Wash Buffer

Reagent	Final concentration	Amount
RNAscope Wash Buffer (50×)	1×	40 mL
ddH_2_O	N/A	1,960 mL
**Total**	**N/A**	**2,000 L**

Store at RT for up to 1 year.

**Table:** 0.5× Wash Buffer

Reagent	Final concentration	Amount
1× RNAscope Wash Buffer	0.5×	500 mL
ddH_2_O	N/A	500 mL
**Total**	**N/A**	**1,000 mL**

Store at RT for up to 1 year.

**Table:** 1× TBS-T

Reagent	Final concentration	Amount
20× TBS IHC Wash Buffer with Tween 20	1×	50 mL
ddH_2_O	N/A	950 mL
**Total**	**N/A**	**1,000 mL**

Store at 4°C for up to 1 year.

**Table:** 1 M Borate pH 8.5

Reagent	Final concentration	Amount
Borate	1 M	61.83 g
Sodium Hydroxide	0.25 M	10 g
ddH_2_O	N/A	Fill to 1,000 mL
**Total**	**N/A**	**1,000 mL**

Store at RT for up to 5 years.

**Table:** Tyramide Signal Amplification (TSA) Buffer

Reagent	Final concentration	Amount
1 M Borate	0.1 M	1,000 μL
Dextran Sulfate 50% Solution	2%	400 μL
30% H_2_O_2_ Solution	0.003%	1 μL
ddH_2_O	N/A	Fill to 10 mL
**Total**	**N/A**	**10 mL**

Make fresh each use.

**Table:** TSA-Biotin

Reagent	Final concentration	Amount
TSA Buffer		300 μL
C1 Biotin		6 μL
**Total**	**N/A**	**306 μL**

Make fresh each use, can be stored on ice for up to 1 h.

**Table:** TSA-DIG

Reagent	Final concentration	Amount
TSA Buffer		300 μL
C2 DIG		6 μL
**Total**	**N/A**	**306 μL**

Make fresh each use, can be stored on ice for up to 1 h.

**Table:** 10% Bovine Serum Albumin (BSA) w/v

Reagent	Final concentration	Amount
BSA	10% w/v	5 g
ddH_2_O	N/A	50 mL
**Total**	**N/A**	**50 mL**

Aliquot in 10 mL aliquots and store at −20°C for up to 1 year.

**Table:** Staining Wash Buffer

Reagent	Final concentration	Amount
10% w/v BSA	0.1% w/v	10 mL
20× TBS IHC Wash Buffer with Tween 20	1×	50 mL
ddH_2_O	N/A	940 mL
**Total**	**N/A**	**1,000 mL**

Store at 4°C for up to 1 year.

**Table:** 10% Sodium Azide

Reagent	Final concentration	Amount
Sodium Azide	10% w/v	1 g
ddH_2_O	N/A	Fill to 10 mL
**Total**	**N/A**	**10 mL**

Aliquot in 1 mL aliquots and store at −20°C for up to 1 year.

**Table:** Antibody Blocking Buffer

Reagent	Final concentration	Amount
20× TBS IHC Wash Buffer with Tween 20	1×	500 μL
Donkey Serum	5%	500 μL
10% Triton X-100	0.1%	100 μL
10% Sodium Azide	0.05%	50 μL
Ultrapure H_2_O	N/A	8.85 mL
**Total**	**N/A**	**10 mL**

Aliquot in 1 mL aliquots and store at −20°C for up to 1 year.

**Table:** Antibody Diluent

Reagent	Final concentration	Amount
20× TBS IHC Wash Buffer with Tween 20	1×	500 μL
Donkey Serum	5%	500 μL
10% Sodium Azide	0.05%	50 μL
Ultrapure H_2_O	N/A	8.95 mL
**Total**	**N/A**	**10 mL**

Aliquot in 1 mL aliquots and store at −20°C for up to 1 year.

**Table:** Primary Antibody Cocktail

Reagent	Final concentration	Amount
Rabbit anti-CD68	1:100	3 μL
Mouse anti-DIG	1:100	3 μL
Antibody Diluent	N/A	294 μL
**Total**	**N/A**	**300 μL**

Make fresh each time.

Primary antibodies should be selected based on the user’s study design. Note that anti-hapten (e.g., anti-DIG) is needed to visualize hybridization probes.

**Table:** Secondary Antibody Cocktail

Reagent	Final concentration	Amount
anti-Rabbit Alexa Fluor® 488	1:200	2 μL
anti-Mouse Alexa Fluor® 555	1:200	2 μL
Streptavidin Alexa Fluor® 647	1:200	2 μL
Antibody Diluent	N/A	394 μL
**Total**	**N/A**	**400 μL**

Make fresh each time.

**Table:** Fixation Solution

Reagent	Final concentration	Amount
16% PFA	4%	10 mL
10× PBS	1×	4 mL
ddH_2_O	N/A	26 mL
**Total**	**N/A**	**40 mL**

Store at −20°C for up to 1 year or at 4°C for up to 2 weeks in the dark.

**Table:** 75% Ethanol (v/v)

Reagent	Final concentration	Amount
100% Ethanol	75% (v/v)	750 mL
ddH_2_O	N/A	250 mL
**Total**	**75% (v/v)**	

Store at RT for up to 6 months.

## Step-by-step method details

### Deparaffinization and slide rehydration for antigen retrieval

**Timing: 3 h**

The goal of this section is to remove the paraffin coating on the cell pellet/tissue sections and subsequently rehydrate the cells/tissue. This is followed by an optimized HIER (heat-induced antigen retrieval) step with a specific pH 9.0 buffer to expose both nucleic acid targets for ISH and cross-linked epitopes for antibody recognition, without the need for any protease treatment.

1. Deparaffinization: Place the desired slides with FFPE tissue samples in an oven at 70°C for 1 h. After the paraffin has melted, wash with xylenes 2 × 15 min in glass Coplin jars to remove residual paraffin.

***Note:*** While the slides are baking, prepare the Linear Stainer (step 3) and pre-warm the PT Module to 70°C (step 4). See steps 3 and 4 for their respective conditions.

**Figure 2:**
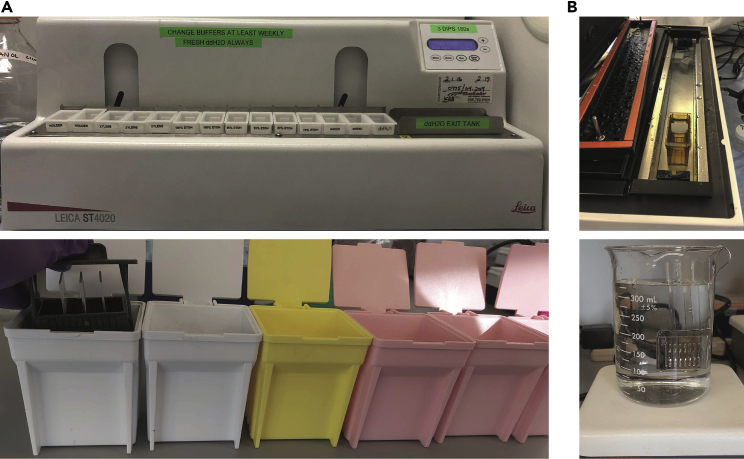
Equipment setup for slide rehydration and HIER (A) Top: Linear Stainer setup for slide rehydration. Each jar should be filled at least three-quarters full to ensure that the tissues are fully submerged at each rinse step. The “holder” jars on the left should be empty and are used to hold extra slides on the Linear Stainer. Bottom: In lieu of a Linear Stainer, one can manually rehydrate tissues using the EasyDip Slide Staining Kit. (B) Top: PT module setup for pre-warming and HIER. The vertical tank is filled with 1× PBS, and the slide chamber is filled with freshly made 1× pH 9 Dako Retrieval Buffer. Bottom: In lieu of a PT module, HIER can be performed by placing the slides in a Staining Tray, submerging the tray in a beaker containing 1× pH 9 Dako Retrieval buffer, and boiling on a hot plate.

***Note:*** In lieu of a Linear Stainer, rehydration can be performed manually using a series of containers such as the EasyDip™ Slide Staining Kit [see Optional Equipment] (Figure 2A, bottom).

2. Rehydration: Rehydrate the slides using a Linear Stainer set to 3 dips and 180s (Figure 2A). The Linear Stainer will automate the rehydration from left to right. Therefore, it is important to prepare the buffers in the following order:
  a. 3 × 180s xylenes washes.
  b. 2 × 180s 100% Ethanol washes.
  c. 2 × 180s 95% Ethanol washes.
  d. 1 × 180s 80% Ethanol [refer to recipe table] wash.
  e. 1 × 180s 70% Ethanol [refer to recipe table] wash.
  f. 3 × 180s ddH_2_O washes.
  g. ddH_2_O holding tank.

**Pause point:** The slides can sit in the ddH_2_O holding tank for up to an hour.

3. HIER: Transfer the slides from the ddH_2_O holding tank to a slide chamber filled with 1× pH 9 Dako Retrieval Buffer [refer to recipe table] and place in the PT module prewarmed to 70°C (Figure 2B, top). Prepare the PT module as follows:
  a. The PT module should be filled with 1× PBS [refer to recipe table]. Ensure that the PBS level is low enough such that it does not spill into the slide chambers while ensuring the PBS level is high enough not to trigger the “low water level” sensor.
  b. The PT module should be set to perform HIER at 97°C for 10 min after preheating to 70°C.
  c. The PT module should be set to control cooling post-HIER to 65°C.

***Note:*** While the PT module gives us the most consistent results, HIER can also be manually performed. For example, slides may be placed in a Staining Tray [see Optional Equipment], submerged in a beaker containing 1× pH 9 Dako Retrieval buffer, and boiled on a hot plate (Figure 2B, bottom).

4. Once the PT module cools to 65°C, remove the slide chamber containing the slides and allow them to equilibrate to room temperature.

**Pause point:** The slides can sit in the 1× pH 9 Dako Retrieval Buffer at room temperature for up to 1 h.

5. Once the slides are cooled to room temperature, rinse in ddH_2_O 2 × 2 min in Coplin jars.
6. Flick the slides to remove excess water and then circle the tissue sections with a hydrophobic pen using a single stroke.

**CRITICAL:** Do not allow tissues to dry out. Perform step 7 as quickly as possible but be careful not to let the hydrophobic pen contact water. Return slides to the ddH_2_O Coplin jars within 1 min to ensure that the tissues stay hydrated. This is also a good chance to check that the hydrophobic circle does not wash off. The integrity of the hydrophobic circle facilitates downstream blocking and staining steps by allowing reagents to be localized to the tissue.

### Blocking and target hybridization

**Timing: 1.5 h + O/N incubation**

The goal of this portion is to maximize on-target binding of the probes, followed by hapten deposition. This is done by blocking endogenous peroxidases with H_2_O_2_, blocking nonspecific biotin binding using a combination of avidin and biotin, and then hybridizing the probes overnight. For the highest reproducibility, we suggest trying this protocol first with RNAscope commercial kit and probes available from ACD Bio, before customizing the assay for your ISH method of choice. We have successfully adapted PANINI to other ISH methods, including custom Oligopaints probes (Beliveau et al., 2012).

**Figure 3:**
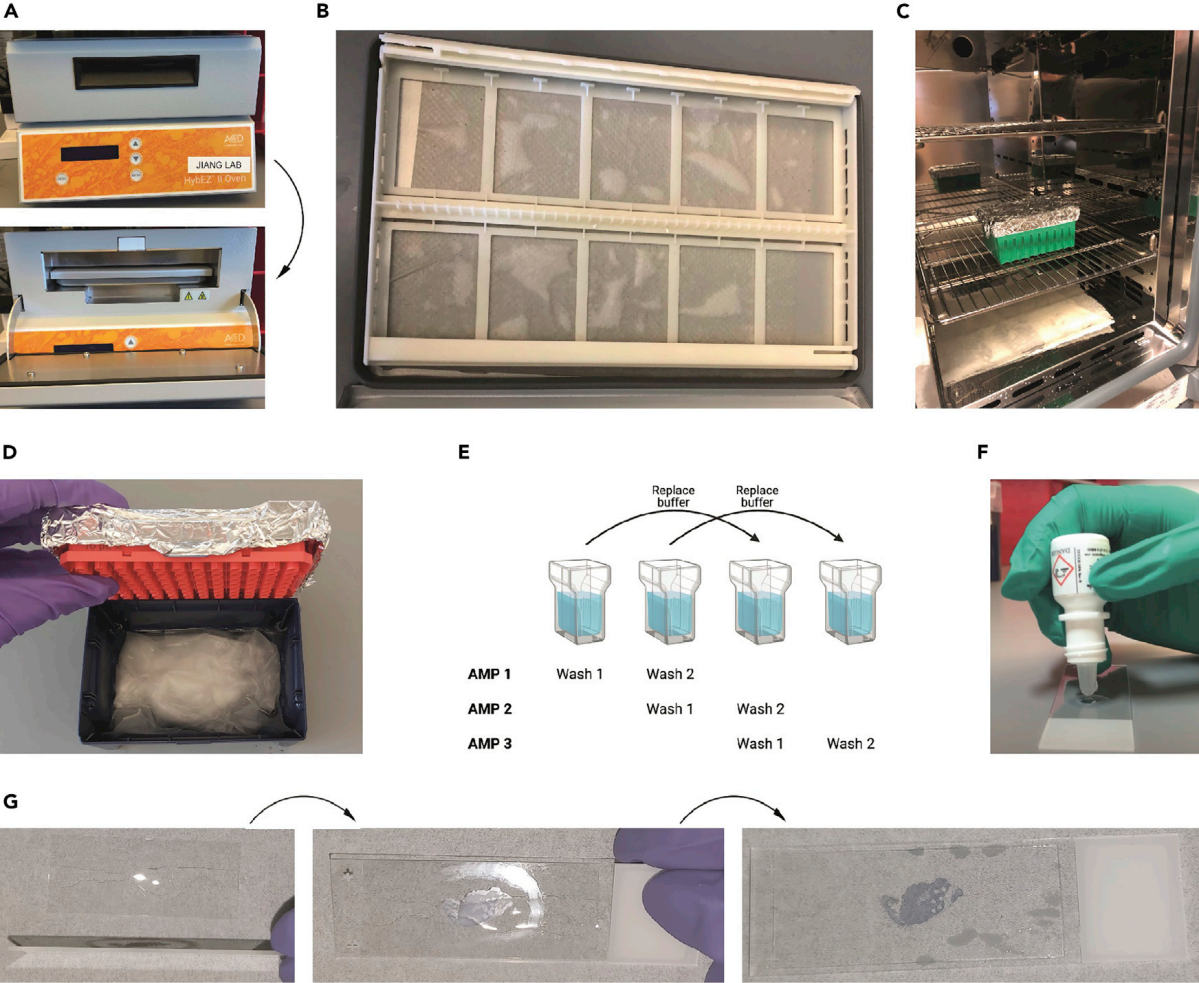
Experimental setup for tissue staining and mounting (A) Manual hybridization system setup for nucleic acid probe amplification. The oven pan fits neatly into the interior. (B) Damp paper towels in the over pan are utilized to maintain humidity during the 40°C amplification steps. (C) A conventional oven and a humidity chamber may be used to substitute the manual hybridization system. (D) Humidity chamber setup for primary antibody staining. The humidity chamber is filled with water and a damp paper towel to maintain humidity during the overnight stain, and the lid is wrapped with aluminum foil to accommodate potential light-sensitive reagents. (E) Setup for slide washing. The second wash of the previous step can be used as the first wash of the new step, for reagent conservation. This setup is applicable to all amplification steps. (F) Staining tissue sections with a dropper. Gently drip enough buffer (1–3 drops) before using the tip to spread the buffer across the tissue without touching it. (G) Mounting the tissue section. First drip the glass coverslip with 40 μL of mounting medium, then place the tissue slide over the No. 1 glass coverslip (22 × 50 mm) with the tissue side facing the coverslip. Excess bubbles are removed as needed.

***Note:*** We highly recommend using equipment from the ACD HybEZ oven system from henceforth. If unavailable, users may substitute this with a different oven and a homemade humidity chamber (Figure 3C), but temperature consistency during the downstream steps cannot be guaranteed. If using a homemade humidity chamber, fill the base of an empty pipette tip box with water and place a paper towel inside to retain moisture. Wrap the lid with aluminum foil so that it can also be used for light-sensitive work (Figure 3D).

***Note:*** Additionally, we use the associated wash trays for all the wash steps; if using alternative equipment, we recommend doing all washes in EasyDip™ Slide Staining Kit (see Figure 2A, bottom). To conserve reagents during the wash steps, users can reuse the second wash of the previous step for the first wash of the following step (Figure 3E). This ensures that the wash buffer can be changed once per wash cycle instead of twice.

7. Transfer slides to the slide holder within the ACD HybEZ oven pan (Figure 3A) and use this to rinse slides 2 × 2 min in ddH_2_O in the ACD HybEZ oven wash trays.
  a. While slides are rinsing, retrieve the Probes from 4°C and place them in a warm water bath (37°C–40°C) such that they are standing with the water line below the cap. This is to prevent any potential salt precipitation in the probes. Do not submerge the Probes in water to minimize possible contamination.
  b. Turn on the oven and prewarm it to 40°C with the tray inside.
    i. The oven tray should have a ddH_2_O-dampened paper towel inside to create humidity and ensure the slides do not dry out (Figure 3B).

8. Drop 1–2 drops of RNAscope H_2_O_2_ [part of the RNAscope Multiplex Fluorescent Detection Reagents v2 kit] on each slide, or more to ensure the tissue is appropriately covered. Incubate for 20 min at 40°C.

***Note:*** An effective way to distribute the RNAscope H_2_O_2_ and other reagents across the entire section is to gently spread the liquid using the tip of the dropper bottle. Ensure the tip does not directly touch the tissue section to avoid contamination and disrupting the tissue (Figure 3F). Readers are referred to Methods video S1. This technique is applicable to all reagents dropped onto the slides throughout this protocol.

**CRITICAL:** The RNAscope H_2_O_2_ should completely cover the tissue and be sufficient to not evaporate during incubation, but also should not overflow over the hydrophobic circle. This applies to all reagents added to the tissue from this point forward.

9. Rinse slides 2 × 2 min in ddH_2_O in the HybEZ Oven wash pans.
  a. During this rinse, leave the oven pan at room temperature to cool.
10. Add 1–2 drops of Avidin Block [see Critical Commercial Assays] to each slide and incubate in the oven pan at room temperature for 15 min.
11. Rinse slides 2 × 2 min in ddH_2_O.
12. Add 1–2 drops of Biotin Block [see Critical Commercial Assays] to each slide and incubate in the oven pan at room temperature for 15 min.

***Note:*** If users choose to use a different hapten instead of biotin, steps 9–12 can be skipped.

13. Rinse slides 2 × 2 min in ddH_2_O.
  a. During this rinse, prewarm the pan in the oven.
  b. Double-check the oven is still set to 40°C.
14. Add 1–2 drops of the aliquoted nucleic acid probes [see Probes and Antibodies] to each slide. For example, here we combine two different probes to target SIV DNA and SIV RNA [see Probes and Antibodies].
  a. If using a different set and/or number of probes, combine them according to the manufacturer’s specifications.
  b. Be sure to gently mix the probes well before dropping them onto the tissue.
  c. Incubate overnight at 40°C in the dark, with the slide containing the probes exposed inside the humidity chamber. In our experience, this allows for a cleaner result when compared to using a coverslip. Ensure that the paper towels are still wet, so that the pan is humid enough to prevent the probes from evaporating overnight.
  d. This is a good step to switch over to any customized probes or alternative ISH methods. Please adjust the annealing temperature and time according to your optimized assays. For instance, our optimized Oligopaints probe hybridization occurs at 42°C.

**Pause point:** Probes should hybridize for at least 4 h and up to 48 h. In our experience, an overnight hybridization (15–18 h) works well.

Methods video S1. A video demonstration of dropping and spreading the reagents on a slide, related to step 8 and all subsequent steps that use a dropper bottle

### Probe amplification and primary antibody binding

**Timing: 5.5 h + O/N incubation**

This section of the protocol describes the series of amplification steps to boost the signal of the DNA and RNA targeting probes by using RNAscope reagents [see Critical Commercial Assays]. The first three amplification steps occur concurrently, but the subsequent amplifications are split in a channel-specific manner for Tyramide Signal Amplification (TSA) and hapten deposition. Here, we use biotin and DIG for viral DNA and RNA respectively, but users may choose to use different haptens.

15. Remove Amps 1–4 [part of the RNAscope Multiplex Fluorescent Detection Reagents v2 kit] from 4°C to room temperature.
  a. Place Amp1 bottle in a water bath at 37°C. Do not submerge the probes in water to minimize contamination risks.
  b. Prepare an ice bucket to store the Amps after their respective applications.
16. Remove slides from the 40°C oven and wash 2 × 2 min in 0.5× Wash Buffer [refer to recipe table].
  a. Be sure to note the start and stop time of incubation.
17. Add 1–2 drops of Amp1 to each slide and then incubate at 40°C for 30 min.
  a. Place the Amp2 bottle in the front nook of the oven during the incubation to pre-warm it.

***Note:*** If using an alternative oven, be sure to prewarm Amp2 and subsequent Amp bottles that require preheating in a water bath set to 40°C.

18. Wash 2 × 2 min in 0.5× Wash Buffer.
  a. During the wash, place the pan and Amp2 back in the oven to keep them warm.
19. Add 1–2 drops of Amp2 to each slide and then incubate at 40°C for 15 min.
  a. Place the Amp3 bottle in the front of the oven during the incubation to pre-warm it.
20. Wash 2 × 2 min in 0.5× Wash Buffer.
  a. During the wash, place the pan and Amp3 back in the oven to keep them warm.
21. Add 1–2 drops of Amp3 to each slide and then incubate at 40°C for 30 min.
  a. Place the Amp4 HRP-C1 bottle [part of the RNAscope Multiplex Fluorescent Detection Reagents v2 kit] in the front of the oven during the incubation to pre-warm it.
22. Wash 2 × 2 min in 0.5× Wash Buffer.
  a. During the wash, place the pan and V2 Amp4 back in the oven to keep them warm.
23. Add 1–2 drops of V2 Amp4 to each slide and then incubate at 40°C for 15 min in the dark.
  a. Remove Amp5 and Amp6 bottles [part of the RNAscope 2.5 HD Detection Reagent- BROWN] from 4°C and allow them to equilibrate to room temperature. Do NOT prewarm these reagents to 40°C or the reaction will not be specific.
24. Wash 2 × 2 min in 0.5× Wash Buffer.
  a. During the wash, leave the pan uncovered at room temperature to cool.

***Note:*** If not using the ACD Bio HybEZ oven system, leave the humidity chamber out of the oven during the steps in which the pan is left uncovered at room temperature.

The following three steps use additional reagents from the RNAscope 2.5 HD Detection Reagent- BROWN kit, which further boosts the TSA reaction required for robust visualization of DNA targets. This step may be skipped if only abundant RNA is intended for visualization. These reagents should NOT be pre-warmed to 40°C to avoid assay failure.

25. Add 1–2 drops of brown kit Amp5 [part of the RNAscope 2.5 HD Detection Reagent- BROWN] to each slide and then incubate at room temperature for 30 min in the dark.
26. Wash 2 × 2 min in 1× TBS-T [refer to recipe table].
  a. Keep the pan at room temperature.
27. Add 1–2 drops of brown kit Amp6 [part of the RNAscope 2.5 HD Detection Reagent- BROWN] to each slide and then incubate at room temperature for 15 min in the dark.
  a. During the incubation, prepare the TSA buffer [refer to recipe table] and TSA reagents (TSA-Biotin and TSA-DIG) [refer to recipe table].

***Note:*** Make the TSA buffer fresh and do not add 30% H_2_O_2_ Solution until immediately before use. TSA DIG can be placed on ice until needed.

28. Wash 2 × 2 min in 1× TBS-T.
29. Add 50–100 μL of the prepared TSA-Biotin to each slide and then incubate at room temperature for 15 min.
  a. In general, 50–100 μL would suffice to cover the entire tissue.
30. Wash 2 × 2 min in 0.5× Wash Buffer.
31. Add 1–2 drops of HRP-Blocker [part of the RNAscope Multiplex Fluorescent Detection Reagents v2 kit] to each slide and then incubate at 40°C for 15 min.
  a. HRP-Blocker should be kept on ice.
  b. Place Amp4 HRP C2 [part of the RNAscope Multiplex Fluorescent Detection Reagents v2 kit] in the front of the oven during the incubation to pre-warm it.

**CRITICAL:** This step will fail at room temperature; make sure the incubation is at 40°C. Ensure the slides are covered as the reaction is light-sensitive.

32. Wash 2 × 2 min in 0.5× Wash Buffer.
  a. During the wash, place the pan and Amp4 HRP C2 back in the oven to keep them warm.
33. Add 1–2 drops of Amp4 HRP C2 to each slide and then incubate at 40°C for 15 min in the dark.
34. Wash 2 × 2 min in 0.5× Wash Buffer.
35. Add 50–100 μL of the prepared TSA-DIG to each slide and then incubate at room temperature for 15 min in the dark.

**CRITICAL:** Ensure the slides are covered as the reaction is light-sensitive.

36. Wash 2 × 2 min in 1× TBS-T.
37. Remove slides and wash 2 × 5 min in Staining Wash Buffer [refer to recipe table] in a Coplin jar.
38. Transfer slides to a humidity chamber and add ∼100 μL of Antibody Blocking Buffer [refer to recipe table] to each slide while ensuring the tissues are fully covered. Incubate for 1 h at room temperature.
  a. If the slides are to be used with a multiplexed imaging modality, such as the CODEX and MIBI, this step can be streamlined into their respective antibody blocking steps.

**Pause point:** Slides can be left to block for up to 3 h.

39. Prepare the Primary Antibody Cocktail [refer to recipe table] while waiting for the block to complete. Once complete, add 100 μL of the primary antibody cocktail per slide.
  a. The antibodies used here have been optimized at a concentration of 1:100 antibody to Antibody Diluent [refer to recipe table].

***Note:*** Users should carefully select primary antibodies based on the study design and validate via immunohistochemistry (IHC) on FFPE tissue or cell pellet sections. However, anti-hapten antibodies (e.g., anti-DIG) must be included as it is needed to visualize hybridization probes.

### [Optional] Secondary antibody staining

**Timing: 2 h**

The goal of secondary antibody staining is to label the bound primary antibodies with fluorescent-conjugated antibodies for imaging using a standard fluorescence microscope. This step should be skipped for other high-plex imaging modalities.

***Note:*** The need for secondary antibody staining depends on the primary antibody, sample type, and imaging modality. For instance, it is not needed if the slides are to be imaged with a high-plex imaging modality, such as the CODEX and MIBI.

40. Remove slides from the humidity chamber and wash 2 × 5 min in Staining Wash Buffer.
41. Prepare the Secondary Antibody Cocktail [refer to recipe table] during the wash.
  a. The secondary antibodies used here are diluted at a 1:200 dilution in Antibody Diluent.
42. Add ∼100 μL Secondary Antibody Cocktail per slide and incubate at room temperature for 1 h in the humidity chamber.
43. Wash 2 × 5 min in Staining Wash Buffer in Coplin jars.
44. Place slides into a third Coplin jar containing 30 mL of Staining Wash Buffer and 5 μL of Hoechst, and then allow slides to incubate for 5 min in the dark.
45. Rinse slides for 2 min in Staining Wash Buffer.

**Pause point:** Slides can be stored in Staining Wash Buffer for up to 1 h at room temperature, or overnight at 4°C.

46. Mount a No. 1 coverslip (22 × 50 mm) [see Key Reagents Table] over the slides using ∼40 μL Antifade Mounting Medium [see Key Reagents Table] with DAPI and seal the coverslip with clear nail polish (Figure 3F).

**Pause point:** Slides can be stored in the dark at 4°C for up to 2 weeks, or for months in −20°C.

47. Image slides on a fluorescent microscope.

## Expected outcomes

**Figure 4:**
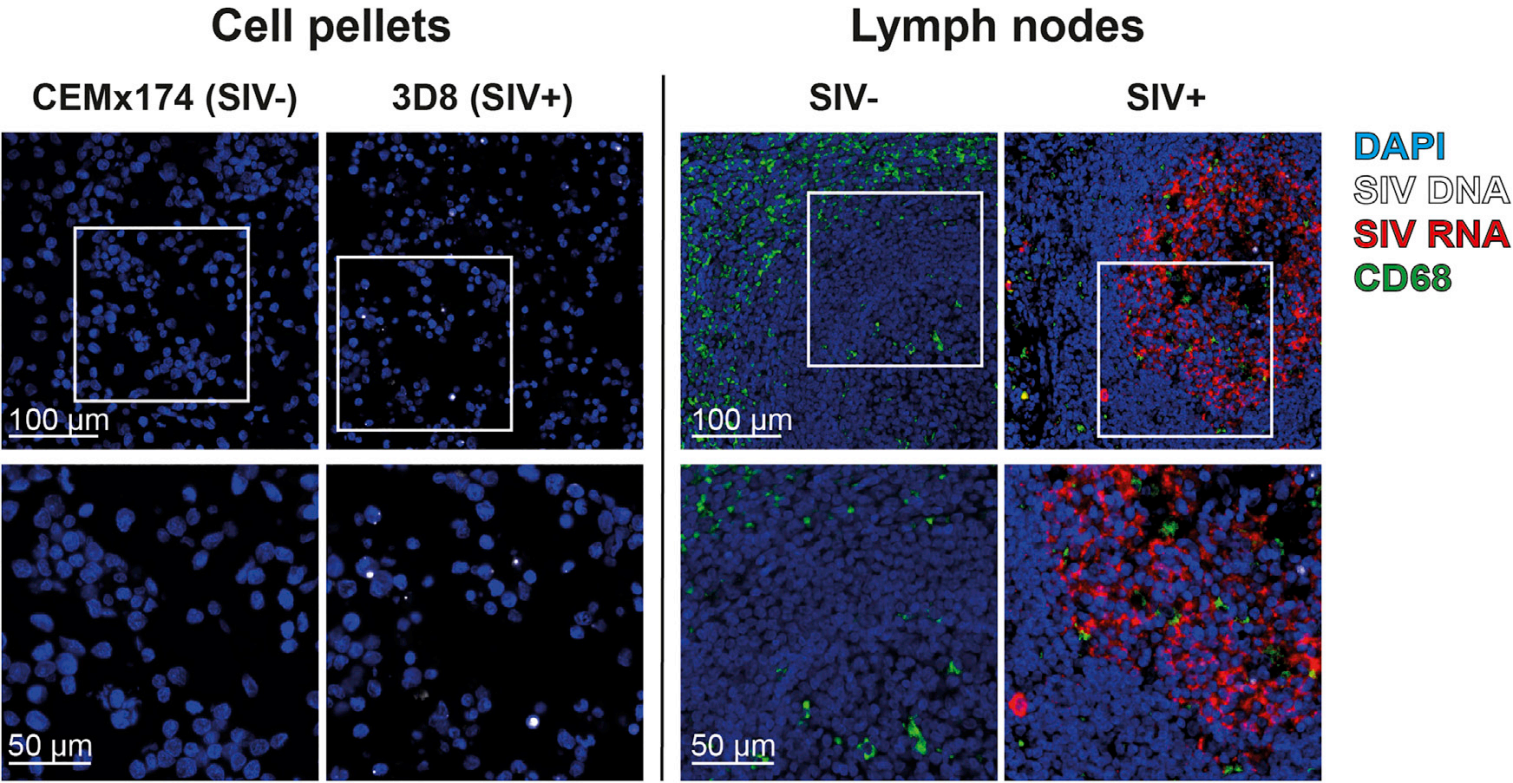
Three-marker PANINI staining in SIV^+^/SIV^-^ cell pellets and lymph nodes Left: Comparison between CEMx174 (SIV^-^, negative control) and 3D8 (SIV^+^, positive control) cell pellets. Right: Comparison between SIV^-^ and SIV^+^ lymph nodes.

Our protocol illustrates how PANINI staining effectively enables tissues to be concurrently stained with nucleic acid probes and antibodies, thereby visualizing DNA, RNA, and proteins simultaneously. Here, we performed PANINI staining on 3D8 (SIV^+^) and CEMx174 (SIV^-^) control cell pellets, as well as SIV-infected and healthy control lymph nodes, to demonstrate PANINI’s sensitivity and robustness (Figure 4).

When comparing 3D8 and CEMx174 (SIV^-^) cells, at 200× magnification, we see that 3D8 cells were positive for SIV DNA (white spots) and RNA (red spots) while CEMx174 cells were negative for both. Importantly, at 600× magnification, we see that SIV DNA spots in 3D8 cells were exclusively localized within the cell nuclei (blue), which is concordant with the location of integrated proviral DNA. As 3D8 cells contain only a single copy of integrated proviral DNA per cell (Deleage et al., 2016; Nishimura et al., 2009), our results clearly show PANINI’s sensitivity to single genomic events. For a more detailed analysis of the assay sensitivity, we refer readers to Jiang et al. (2022).

When comparing SIV^+^ and SIV^-^ lymph nodes, at 200× magnification, we found that SIV infected cells and virions are enriched in the follicles, and, at 600× magnification, detailed productive replication events at the single-cell level. These results show that even a simple three marker PANINI staining could illustrate how SIV manifests in lymph nodes within the native tissue context. Therefore, we envision that the ability to scale PANINI with multiplexed tissue imaging modalities will be very promising in the future.

## Limitations

While we only demonstrate here the application of PANINI to archival rhesus macaque and cell pellets from various human-derived cell lines, our laboratory has been successful in applying PANINI to other host and viral nucleic acids (Jiang et al., 2022). We anticipate PANINI to remain broadly applicable across multiple species and differently preserved tissue samples. The streamlined workflow here is an effective avenue for the validation of novel protein and nucleic-acid targeting reagents.

A key limitation of PANINI is the number of “channels” currently detectable for nucleic acids. We have successfully validated up to four antibody-hapten pairs to date, although future possibilities will likely be possible with novel tyramide chemistry.

While we note the use of the commercially available RNAscope reagents in this protocol, PANINI can also be performed with alternative ISH methods, including Oligopaints (Beliveau et al., 2012), SABER (Kishi et al., 2019), and Hybridization Chain Reaction (Choi et al., 2018), thereby expanding its practical usage to the user’s preferred choice of nucleic acid visualization.

## Troubleshooting

### Problem 1

No ISH signal.

No nuclear (DNA) or cytosolic (RNA) signals on their corresponding ISH channels.

ISH signals should appear punctate and localized at their appropriate cellular locations: DNA-ISH should be nuclear, and RNA-ISH should be mostly cytosolic. In addition, RNA is generally significantly more abundant than DNA. Refer to Figure 4 for an example of the appearance of DNA-ISH and RNA-ISH signals.

### Potential solution

The probes may have been washed off across the “Probe Amplification” steps.

Solution: Perform gentler washes by using 1× Wash Buffer instead of 0.5× Wash Buffer.

The anti-hapten dilutions (streptavidin and anti-DIG antibody) were too low.

Solution: Use a higher anti-hapten primary and/or secondary antibody concentration.

Too few probes were used against the target of interest.

Solution: Increase the number of probes against the target of interest and use a higher concentration of TSA reagents. Users may also consider using another ISH amplification method.

Reagent issues with the probe amplification kit.

Solution: Check the expiration date of the amplification kit and consider using a fresh kit and associated reagents.

Issues with the tyramide reagents.

Solution: Ensure that the tyramide reagents are stored according to manufacturer recommendations and prepare fresh TSA buffers each time. Users may also consider replacing the 30% H_2_O_2_ Solution stock as H_2_O_2_ has a relatively short half-life when stored inappropriately.

### Problem 2

No protein target signal.

No signal on the channels corresponding to the protein marker.

Protein signals should exhibit a wide linear dynamic range and localize to their appropriate cellular locations (e.g., nuclear, membrane, cytoplasm). In addition, protein staining patterns may be highly dependent on tissue preservation and type.

### Potential solution

Primary Antibody Cocktail concentration against the protein target(s) was too low.

Solution: Increase primary antibody concentrations.

Antibody clones are not compatible with the tissue fixation conditions.

Solution: Try an alternative clone or adjust retrieval conditions. Perform a positive control in which a well-optimized antibody against another target is tested for in the tissue in question, to confirm that poor tissue preservation or storage is not a factor. Using an antibody against an abundant target, such as Pan-Keratin or CD45, would be a good positive control to confirm that the assay is working.

HIER duration is not optimized for epitopes.

Solution: Increase the 97°C HIER duration for up to 40 min, and try alternative HIER buffers, including citrate-based ones.

Protein of interest is not expressed in the tissue.

Solution: Test primary antibody against positive and negative control tissues.

### Problem 3

Unspecific signal.

The intended signals appear but are not consistent with their expected appearance, such as non-punctate ISH signals and unexpected protein staining patterns. Refer to Figure 4 for an example of proper ISH and protein staining patterns.

### Potential solution

Washes are not long and/or harsh enough.

Solution: Increase the length of time for each wash by 1 min and/or decrease the Wash Buffer salt concentration (e.g., by diluting it from 0.5× to 0.4×) for harsher wash conditions. Make sure that the tissues are fully covered during the washes.

Tissue dried out during the protocol.

Solution: Do not let tissues dry out between steps to prevent the buildup of nonspecific signals. Make sure that the tissues are fully covered during the washes, ensure that the slides are quickly moved between washes, and draw the hydrophobic barrier as quickly as possible.

TSA assay has non-specific signals.

Solution: Increase the duration and concentration of H_2_O_2_ blocking to eliminate background. Users may also consider further optimizing the concentration of TSA reagents.

Unspecific ISH or protein-targeting antibody probes.

Solution: Confirm probe specificity by using positive and negative control cell pellets. In addition, for more batch-to-batch consistency, we recommend using monoclonal antibodies.

## Resource availability

### Lead contact

Further information and requests for resources and reagents should be directed to and will be fulfilled by the lead contact, Sizun Jiang, sjiang3@bidmc.harvard.edu.

### Materials availability

No new materials were generated for the purpose of this protocol.

## Data Availability

No datasets and code were generated for the purpose of this protocol.
